# Visualizing Viral Infection In Vivo by Multi-Photon Intravital Microscopy

**DOI:** 10.3390/v10060337

**Published:** 2018-06-20

**Authors:** Xaver Sewald

**Affiliations:** Max von Pettenkofer Institute & Gene Center, Virology, National Reference Center for Retroviruses, Faculty of Medicine, LMU München, 80336 Munich, Germany; sewald@mvp.uni-muenchen.de; Tel.: +49-89-2180-78136

**Keywords:** intravital microscopy, multi-photon, virus infection, HIV, murine leukemia virus, MLV, pseudorabies virus, PRV

## Abstract

Viral pathogens have adapted to the host organism to exploit the cellular machinery for virus replication and to modulate the host cells for efficient systemic dissemination and immune evasion. Much of our knowledge of the effects that virus infections have on cells originates from in vitro imaging studies using experimental culture systems consisting of cell lines and primary cells. Recently, intravital microscopy using multi-photon excitation of fluorophores has been applied to observe virus dissemination and pathogenesis in real-time under physiological conditions in living organisms. Critical steps during viral infection and pathogenesis could be studied by direct visualization of fluorescent virus particles, virus-infected cells, and the immune response to viral infection. In this review, I summarize the latest research on in vivo studies of viral infections using multi-photon intravital microscopy (MP-IVM). Initially, the underlying principle of multi-photon microscopy is introduced and experimental challenges during microsurgical animal preparation and fluorescent labeling strategies for intravital imaging are discussed. I will further highlight recent studies that combine MP-IVM with optogenetic tools and transcriptional analysis as a powerful approach to extend the significance of in vivo imaging studies of viral pathogens.

## 1. Introduction

Imaging studies under experimental in vitro conditions revealed exciting details of the replication cycle of many different viruses. Virus entry through endocytosis and fusion, intracellular trafficking and nuclear import, and assembly and release of viral particles has been extensively studied in vitro using cancer cell lines and primary cells. Thereby, fluorescence microscopy played an important role as the visualization of viral and cellular proteins by fusion to fluorescent proteins or immunostaining allowed researchers to define subcellular structures that are critical for various steps of the viral life cycle. In particular, live cell imaging using confocal laser-scanning or spinning-disk microscopy provided valuable dynamic information in three dimensions (3D) at the subcellular level of in vitro cultured cells [1,2]. In return, viruses such as the model virus simian virus-40 allowed researchers to unravel basic concepts in cell biology such as caveolar endocytosis, endosomal trafficking, and cellular transformation [3,4,5,6]. More recently, novel imaging techniques such as super-resolution fluorescence microscopy [7,8] and cryo-electron tomography [9,10,11,12,13] revealed further details at high resolution or even at the molecular level using single-molecule fluorescence resonance energy transfer (FRET) imaging [14,15].

Only a few imaging techniques, however, allow the study of biological events within the complex tissue environment of a living organism. Whole body bioluminescence in vivo imaging is a powerful approach to follow cells of interest over long time periods within intact tissue of the same organism. Tumor cells or cells infected by luciferase-encoding reporter viruses have been visualized within different tissues even deep inside a living organism [16,17,18,19,20,21,22,23,24]. Recently, substrates and luciferases have been improved for in vivo applications. More efficient substrate-enzyme combinations with red-shifted emission spectra enabled researchers to visualize very small cell numbers even in real-time [25,26,27]. Although bioluminescence imaging has been combined with micro-computed tomography to acquire 3D information of the luminescence signal the resolution remains restricted to the macroscopic scale of organs. For in vivo imaging studies with cellular and subcellular resolution intravital fluorescence microscopy has been applied. Because of the photophysical properties of the tissue intravital imaging using multi-photon excitation of fluorophores is the method of choice to visualize and quantify biological processes. The dynamics of cells, the subcellular distribution of proteins but also cell signaling events have been analyzed within intact tissue (for review see [28,29]). In addition, multi-photon intravital microscopy (MP-IVM) has been used to directly visualize viral pathogens in different tissues. For example, fusion of the green fluorescent protein (GFP) to the structural capsid protein Gag of the mouse retrovirus MLV allowed researchers to observe trans-infection events and virological synapses in vivo [30,31]. More recently, STED microscopy using high-pulsed laser for multi-photon excitation has been applied to visualize neuronal cells activity and connectivity in brain slices and even intact brain tissue of living mice at nanometer resolution [32,33].

## 2. Multi-Photon Excitation Microscopy

**Figure 1:**
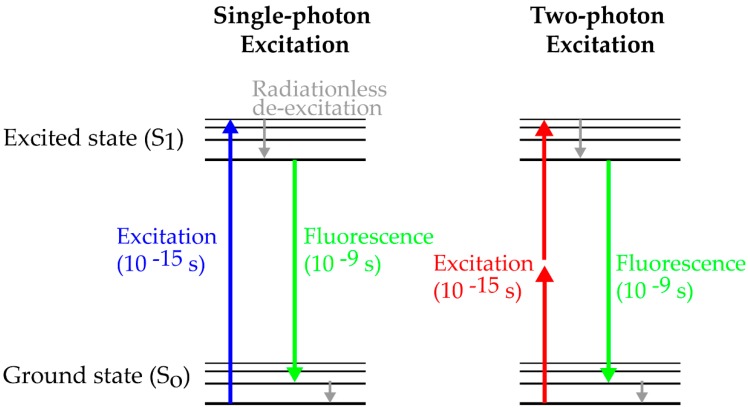
Simplified Perrin-Jablonski scheme for single-photon and two-photon excitation. Fluorophores reach the excited state after absorption of a single photon or of two photons within femtoseconds. After de-excitation and emission of fluorescence light, the molecule reaches the ground state within nanoseconds. Importantly, fluorescent emission is identical after single- and two-photon excitation.

The theoretical framework of multi-photon excitation originates from work by Göppert-Mayer in the 1930s [34]. The concept describes the absorption of two photons of identical frequencies to excite a molecule from the ground state to a higher energy state. The energy difference between the molecular states is equal to the sum of the energies of both photons. Alternatively, a molecule can also reach the high energy state after absorption of a single photon with the equivalent amount of energy. Considering the excitation of the fluorescent protein GFP, absorption of a single photon with 480 nm leads to the excitation of GFP reaching the high energy state, followed by non-radiative de-excitation and subsequent emission of a photon with less energy, i.e., longer wavelength (Figure 1). For two-photon excitation, a GFP molecule must absorb two photons of half of the energy (i.e., double wavelength) within femtoseconds for excitation and subsequent light emission (Figure 1). The absorption of a single, lower energy photon with 960 nm is not sufficient to completely excite the GFP molecule. Thus, the GFP molecule will return within femtoseconds to the ground state after absorption without photon emission. To reach the high energy state it requires two photons coinciding in time and space. Since only laser light sources can provide the necessary photon density at the focal plane to support two-photon excitation of a molecule it took several years until the development of high-pulsed lasers to experimentally prove the concept of two-photon absorption in the 1960s [35]. Finally, the pioneering work of Denk et al. combined an infrared mode-locked laser with a point scanning microscope to proof imaging of biological samples using 2-photon excitation [36].

**Figure 2:**
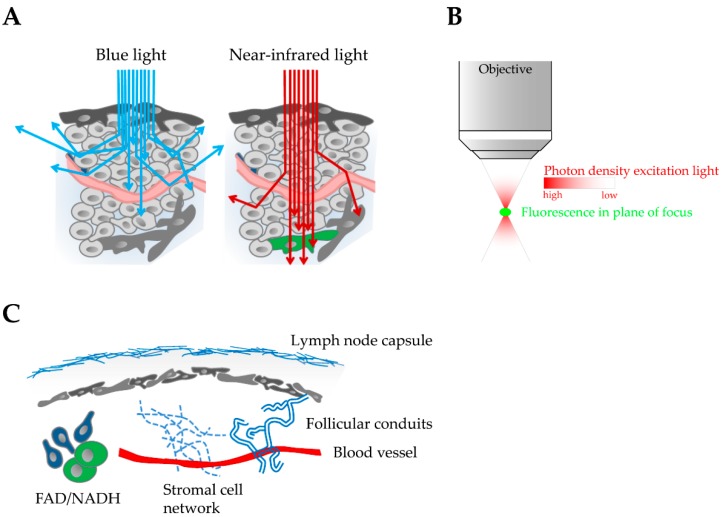
Properties and advantages of multi-photon excitation for in vivo imaging in complex tissue. (**A**) Blue light used for single-photon excitation is absorbed and scattered by different tissue components. In contrast, near-infrared light can penetrate deep into tissue for multi-photon excitation of fluorescent proteins (green cell). (**B**) Intrinsic optical sectioning effect during multi-photon excitation. Sufficient photon density for fluorophore excitation is only reached in the plane of focus. (**C**) High energy multi-photon excitation generates second harmonic signals to visualize tissue structures such as collagen of the lymph node capsule, blood vessels, follicular conduits, and the stromal cell network of lymph tissue. Multi-photon excitation also supports the analysis of the cellular metabolic state by visualizing the cellular co-factors FAD and NADH.

The following photo-physical properties make multi-photon excitation the preferred technique to image biological events under physiological conditions in complex tissue. First, near-infrared light used for multi-photon excitation of fluorophores can penetrate deeper into tissue than light with shorter wavelength (Figure 2A). Absorbance of photons by heme-containing proteins such as oxygenated hemoglobin, pigment proteins such as melanin, water and lipids is minimal and the amount of light scattering is significantly reduced at wavelengths in the infrared spectrum compared to single-photon excitation with light of shorter wavelength [37,38,39]. Since each tissue such as brain, lymph nodes and spleen has a specific composition, penetration depths of MP-IVM can be diverse and even change within the same tissue. For example, brain tissue allows fluorophore excitation of up to 1 mm depth whereas image quality of the liver and spleen deteriorates below 100 µm because of the high density of heme-containing proteins particularly of red blood cells. Second, fluorophore excitation is exclusively achieved in the focal plane where the photon density is sufficiently high for two photons to interact simultaneously (within femtoseconds) with the same fluorescent molecule (Figure 2B). Consequently, an intrinsic optical sectioning effect is achieved with almost no out-of-focus light and reduced phototoxicity and photobleaching. Because the entire signal arises from the focal plane of interest, no pinhole is required in front of the detection unit. Therefore, non-descanned detectors with a simplified optical path for the efficient detection of emission light can be used in multi-photon microscopy. Third, the intense short pulsed laser in multi-photon microscopy allows the excitation of cellular molecules such as intracellular NADH/FAD that can be used to determine the metabolic rate of cells in vivo (Figure 2C) [40,41,42,43]. In addition, tissue molecules that lack a center of symmetry can convert light to its second harmonic, a process also called frequency doubling, at twice the frequency and half the wavelength [44]. This phenomenon is called second harmonic generation (SHG) and depends on very high light intensities such as are given by a pulsed laser. Since the process depends on the square of the intensity, it will be focal plane selective in the same way as two-photon excitation of fluorescence molecules. Unlike common fluorescent proteins, the signal derived from SHG does not bleach or blink and cannot saturate with increasing excitation intensity. Because of its molecular structure, collagen is the strongest source of second harmonic signal in animal tissue and provides a valuable tool for 3D imaging of structural proteins in biological samples [45,46]. Therefore, SHG is of big interest for intravital imaging studies because it allows the visualization of anatomical landmarks such as the lymph node capsule, blood vessels, stromal cell networks and follicular conduits (Figure 2C).

## 3. Experimental Systems for Imaging Viral Infections

Many experimental models with various levels of complexity have been used to visualize infections with many different viruses. Homogenous in vitro cultures of a single cell type or combinations of two different cell types have been studied by live cell confocal fluorescence microscopy using spinning-disk or laser-scanning setups [1]. For instance, cell-to-cell transmission of the retroviruses human immunodeficiency virus (HIV) and murine leukemia virus (MLV) [47,48,49,50,51] but also neurotropic viruses such as Pseudorabies virus (PRV) [52] could be followed in vitro in real time, providing important dynamic information at the subcellular level in 3D. More complex experimental conditions consisting of a mixture of primary cells derived from homogenized host tissue have been used to study virus infection under more physiological conditions. Human ex vivo organ cultures such as human lymphoid aggregated cultures (HLAC) of tonsil tissue [53] and ex vivo cultures of non-inflamed spleen tissue or thymus have been used to study HIV infection and pathogenesis [54]. Recently, in vitro 3D cultures built from cell lines and primary cells have been established to study specific functional tissue compartments. The choriocarcinoma-derived JEG-3 cell line with morphological and functional similarities to primary human syncytiotrophoblasts has been used as a 3D culture model to study microbial infection of placental tissue [55]. In addition, organotypic cell culture models grown in 3D using a rotating wall vessel bioreactor have been developed to study Coxsackievirus B infection of the gastrointestinal epithelium as well as Zika virus infection of the blood-brain-barrier in vitro [56,57]. Furthermore, stem cell-derived human intestinal organoids resembling the small intestine have been developed to study viral infections [58]. Enteroid cultures allowed infection studies with human rotavirus, human norovirus, and enterovirus in vitro [59,60,61].

Although cell culture models and organotypic ex vivo cultures provide important insights into viral infections, direct in vivo studies including intravital microscopy are crucial for our understanding of virus dissemination and pathogenesis. Small animal models but also non-human primates have been extensively used to study viral pathogenesis and the host immune response to viral infection. Recently, MP-IVM was applied to visualize viral infections in tissues such as lymph nodes, CNS and the spleen of different mouse models including humanized mice [30,31,62]. Since each tissue compartment is unique with respect to physiology, anatomical structure and host immune response, certain critical aspects can only be recapitulated under in vivo conditions. First, each tissue consists of a specific cell composition, under steady-state and inflammatory conditions, which can hardly be copied with current in vitro models. Viruses may require tissue-specific cell populations within niches to establish infection and propagate the infection. Th17 cells within the vaginal mucosa of macaques have been shown to be primarily targeted by SIV [63] and lymph-derived MLV was shown to establish infection at secondary lymph nodes by specifically infecting the B-1 cell subpopulation [31]. Also, murine norovirus has very recently been shown to target virus receptor-expressing tuft cells, a rare type of epithelial cells that protrude into the intestine [64] and use this niche as a reservoir to escape the immune response for fecal shedding and persistence [65,66]. Second, intrinsic cellular properties depend on cues provided by the tissue environment that are important for the cell´s physiological function but also viral pathogenesis. For example, motility of leukocytes and the polarity of neurons are cell type-specific features that are crucial for the efficient systemic spread of HIV and neurotropic viruses, respectively. Blocking lymphocyte egress from secondary lymphoid tissue using the sphingosine-1-phosphate receptor antagonist FTY720 reduced systemic dissemination of HIV in a humanized mouse model indicating a role of HIV-infected migratory T cells in the systemic spread of HIV [62]. Rabies virus uses directed axonal transport to ensure neuroinvasion and virus replication in the central nervous system [67]. Third, specific biophysical parameters of tissues such as pore size, stiffness, and charge of the extracellular matrix (ECM), adhesive properties of the cellular environment and shear forces of blood and lymph flows are likely important determinants of virus replication and pathogenesis in vivo. MP-IVM of HIV-infected humanized mice revealed syncytia formation of infected CD4+ T cells in the lymph node and spleen of infected mice [62]. Interestingly, in vitro experiments using a mix of ECM proteins, derived from an in vitro-cultured mouse sarcoma cell line, show formation of HIV-induced syncytia depending on the stiffness of the surrounding 3D environment, thus indicating that ECM properties might contribute to virus pathogenesis in vivo [68].

In summary, a combination of all cues may dictate tissue-specific infection rates and dissemination routes emphasizing the importance of in vivo studies for infection research.

## 4. Challenges for Intravital Imaging

MP-IVM is increasingly used to study dynamic processes at the cellular and subcellular level occurring in their natural environment in vivo. A major challenge for in vivo imaging studies thereby is to maintain experimental conditions as close as possible to the physiological state. The following paragraph summarizes important aspects concerning the preparation of the experimental animal and strategies for fluorescent cell labeling, both critical for successful IVM studies.

### 4.1. Animal Preparation for Short- and Long-Term Intravital Imaging

Imaging biological processes in vivo often requires specific preparation of the animal to expose the tissue of interest. The extent of manipulation is influenced by the accessibility of the tissue and ranges from simple fixation of the animal to complicated microsurgical procedures to expose the relevant organ. Although surgical preparations are inevitably associated with tissue irritation or trauma, the extent of damage depends on the tissue location and the experience of the surgeon. For example, imaging at the skin requires minimal specimen preparation although chemical or mechanical hair removal can cause local irritations that influence tissue physiology [69]. In contrast, complex surgeries are necessary to access other tissues such as lymph nodes, lung, and brain, that inevitably cause tissue trauma with a possible impact on the experimental results. As an example, craniotomy for imaging studies of brain tissue may induce cell injury responses leading to a profound proliferation of astrocytes or dendritic spine turnover [70,71].

Quantitative analysis of dynamic processes in vivo strictly depends on the stable acquisition of images over time. Movement artifacts caused by the heartbeat and respiration of the anesthetized animal are a major obstacle even for peripheral tissues since movement is relayed by the body skeleton. Therefore, physical fixation of the animal at specific skeletal points is often necessary to guarantee optimal imaging results with the risk of additional impact on tissue physiology. For example, imaging at the popliteal lymph node and the brain requires fixation of the hind leg and skull, respectively. In contrast, intra-abdominal organs such as the gut and spleen are readily accessible because they can be exteriorized from the body cavity and directly immobilized to external fix points. In this case, wetting and temperature maintenance of the exposed tissue are the most critical factors to control. If the organ of interest exhibits intrinsic movement as part of its physiological function, alternative strategies are applied to reduce movement artefacts. For example, MP-IVM at the lung during infection has been performed using suction and triggering devices that stabilize and synchronize breathing activity with image acquisition [72,73,74,75,76]. Alternatively, drugs can be locally applied in the case of IVM studies of the gut to reduce peristaltic movement of the muscle layers. However, it is important to keep in mind that drugs (including anesthetics) and other reagents that are applied can affect the physiological functions of host tissue. For example, anesthetic-mediated reduction of blood pressure was observed to impair kidney function and second messenger concentration [77,78]. In addition, thermoregulation of the experimental animal is impaired during anesthesia [79]. Since cellular functions such as lymphocyte migration are highly susceptible to core body temperature changes, a heating stage to maintain temperature and temperature surveillance are mandatory. To further support physiological conditions during IVM, anesthesia is ideally supported by cardiorespiratory monitoring using transcutaneous pulse oximeters for non-invasive recordings of oxygen saturation, respiratory rate, and heart rate. Further, hydration of the experimental animal must be maintained through subcutaneous application of depots or continues intraperitoneal administration since anesthetics are mostly administered by inhalation leading to fast dehydration.

Most intravital imaging studies of viral infections have been performed over a short time window, usually several hours of image acquisition. To follow an infection over a longer period, different animals are imaged at each time point. Unfortunately, inter-animal variations and differences in surgical preparations of the animals lead to less comparative data sets with consequences for data interpretation. Long-term repetitive imaging of the same specimen would therefore be an interesting alternative for certain experimental questions. Although the skin, footpad and eye can be imaged repeatedly in a noninvasive manner [80,81,82], imaging of relevant organs during viral infection such as the brain or secondary lymphoid tissue requires special surgical preparation. For repeated imaging of the prepared tissue of interest imaging window techniques have been developed that allow long-term imaging of different organs within the same animal [83]. An abdominal imaging window allows repeated access to organs such as the liver, intestine and kidney for up to 4 weeks [84,85]. Another example is a mammary imaging window that has been applied to follow individual tumor cells expressing photo-switchable proteins in a mammary carcinoma [86]. Window techniques have also been applied to visualize biological events at secondary lymphoid tissues repeatedly over long time periods. The implantation of an internal window chamber allowed the analysis of lymphoma cell influx into inguinal lymph nodes for up to 14 days [87]. Also, an externally positioned chronic lymph node window was developed for longitudinal intravital imaging of the inguinal lymph node in mice without the need for serial surgeries while preserving local blood and lymph flow [88].

In summary, although MP-IVM comes close to study biological effects under natural conditions, researchers must be aware that the physiological status is inevitably influenced by the required procedures. Animal manipulation should always be kept at a minimum and alternative methods should be considered. The quality of acquired data depends strongly on the preparation and monitoring of the experimental animal. A careful and critical data interpretation is always crucial for in vivo imaging experiments.

### 4.2. Labeling Strategies for Intravital Imaging

Short- and long-term imaging using multi-photon excitation is a powerful technique to study viral infections in vivo. The strength of multi-photon microscopy to visualize biological events directly in living tissue is explained in part using light beyond 650 nm for fluorophore excitation. The spectral range between 650–950 nm is described as the first optical window [89] and provides superior conditions for tissue penetration due to minimal absorption and scattering of excitation and emission light.

The identification and molecular engineering of fluorescent proteins that match the first optical tissue window and feature excellent photo-physical properties such as high quantum yields, and extinction coefficients has been crucial for multi-photon imaging studies. Bright and stable fluorophores allow imaging with a sufficient signal-to-noise ratio even at low intracellular concentrations, a prerequisite for optimal image acquisition and data analysis. Extensive mutational strategies improved fluorescent protein gene expression through optimized codon usage, efficient protein folding and fast maturation rates [90]. For multi-color MP-IVM studies the development of large-stoke shift variants of fluorescent proteins has been important [91,92,93], in particular when only a single multi-photon laser source is available. In addition, the development of truly monomeric variants is crucial when fluorescent proteins are expressed as fusion with viral or host cell proteins since oligomerization can perturb their physiological function. Although not detected by standard gel filtration analysis of purified proteins in solution, the formation of oligomers can readily be observed when fluorescent proteins are expressed in cells. A recent visual approach quantified the homo-oligomerization of many different fluorescent proteins under physiologic conditions [94,95]. When expressed as fusion with the transmembrane domain of cytochrome p450, fluorescent proteins are targeted to the membrane of the smooth endoplasmic reticulum (ER). The homo-oligomerization of proteins on opposing ER membranes restructures the tubular ER network into organized smooth ER (OSER) whorl structures. The study revealed that many fluorescent proteins have strong oligomerization properties when expressed at high local protein concentrations in cells. This might resemble the conditions when fluorescent proteins are expressed as fusion with viral proteins such as the retroviral capsid protein Gag. Thus, oligomerization of fluorescent proteins might interfere with the physiological function of the structural capsid protein Gag during retrovirus particle formation and/or maturation.

After careful selection of fluorescent protein combinations for an experiment, strategies to fluorescently label cells must be developed. Cells of interest can be isolated from donor mice and fluorescently labelled in vitro using vital dyes such as CFSE (green) and CMTPX (red) before adoptive transfer into recipient mice for MP-IVM. This approach is very flexible and allows cells of interest to be isolated from specific mouse lines including wildtype, knock-out or transgenic mice. For example, lymphocyte populations such as T cells and B cells can be isolated in great numbers from the spleen of donor mice by negative or positive selection. After in vitro labeling using cell-permeant fluorescent dyes, cells are adoptively transferred by intravenous injection into mice for intravital microscopy. However, pulsed labeling of cells with vital dyes in vitro has limitations for certain MP-IVM studies including long-term imaging. Dye leakage, bleaching or extensive cell division of labeled cells over time results in the dilution of functional cytoplasmic dyes with a significant reduction in the signal-to-noise ratio. Consequently, imaging of subcellular structures with little cytoplasm such as cell protrusions might not be visible by MP-IVM although cell tracking is still possible. Alternatively, cells can be isolated from knock-in mice that ubiquitously express cytoplasmic fluorescent proteins driven e.g., by the chicken β-actin promoter with cytomegalovirus enhancer (CAG) or human ubiquitin C promoter [96]. Many different mouse lines are available that contain reporter genes for cytoplasmic fluorescent proteins such as EGFP and mCherry [97,98]. Due to strong and continuous expression rate of the fluorescent proteins long-term tracking of dividing cells without extensive bleaching is possible. In addition, with a superior signal-to-noise ratio visualization of small cellular structures with low cytoplasm content is feasible. Although the approach to adoptively transfer fluorescent cells of interest into donor mice for functional imaging studies is fast and flexible, some limitations exist that must be evaluated for each experiment. First, adoptive transfer of cells is not applicable for tissue-resident cell populations such as stromal cells of secondary lymphoid tissue, macrophage populations of the spleen, intestinal epithelial cells, or cells of the central nervous system. Especially long-living cells are often part of a tissue-specific cellular network that might not support the integration of adoptively transferred cells. Second, each tissue contains small populations of specific cell types that cannot be isolated in the required number and purity for adoptive transfer into recipient mice. Third, adoptive cell transfer can result in varying numbers of transferred cells homing to the organ of interest, thereby reaching non-physiological cell counts that may result in experimental artifacts. To overcome some of these limitations, cell lineage-specific expression of fluorescent proteins in mice has been established for several cell populations allowing intravital imaging studies of cell subpopulations and resident cells within tissue-specific networks. Leukocyte-specific promoters from the genes *cd11c*, *cx3cr1* and *lysM* have been used to drive expression of fluorescent proteins in dendritic cells (DCs) (YFP), monocytes/DCs (EGFP) and monocytes/neutrophils (EGFP), respectively, eliminating the need for isolation, labelling and adoptive cell transfer [99,100,101]. Neuronal cells in the central nervous system can be visualized in mice that express fluorescent proteins under the control of a modified Thy1 promoter region for specific neuronal expression. In a similar approach, mouse strains with cell lineage-specific expression of the Cre recombinase are available for inducible expression of fluorescent proteins by target gene insertion in the ROSA26 locus using the Cre/LoxP system.

## 5. MP-IVM Studies of Virus Infection

Intravital imaging using multi-photon excitation has been introduced into the fields of neurobiology and immunology very early after the technique was established in the 1990s [102,103,104]. Immunologists developed various approaches to study the dynamics of immune cells at different stages of the immune response under physiological conditions in vivo. MP-IVM has also been applied to visualize the immune response to viral infections in different organs [105]. Adoptive transfer of fluorescent immune cells and the use of reporter viruses allowed the temporal and spatial analysis of a local immune response during infection with different viruses such as herpes simplex virus, vaccinia virus and vesicular stomatitis virus [106,107,108,109]. Although viral pathogens were used, the focus of these studies was on immune cells and the dynamics of their response. Nevertheless, multi-photon imaging studies in neurobiology and immunology paved the way for virologists to visualize viral pathogenesis in vivo. It is, therefore, not surprising that the first model viruses studied have been neurotropic Pseudorabies virus and lymphotropic retroviruses. Using reporter viruses, virologists started to analyze the behavior and consequences of infections at the cellular level as well as the contribution of cellular and viral proteins to observed effects in vivo. In the following section, some of these fascinating results are summarized.

### 5.1. HIV-Infected Cells Can Form Syncytia In Vivo and Contribute to Systemic Spread

In a first study, the infection of humanized mice with HIV reporter virus was monitored in popliteal lymph nodes using MP-IVM [62]. Strikingly, a subset (10–20%) of HIV-infected central memory-like T cells was shown to form syncytia with elongated cell morphology of more than 100 μm lengths. Using GFP fused to a nuclear localization signal connected the Env glycoprotein-dependent formation of multinucleated syncytia to the unusual cell morphology. In addition, HIV-infected human CD4 T cells revealed a reduced migration dynamic compared to uninfected cells. Interestingly, migration of HIV-infected cells was still impaired in the absence of functional Env indicating additional factors, possibly the HIV accessory protein Nef, to be responsible for the decreased T cell motility in vivo. Furthermore, the migratory T cell population was shown to contribute to the systemic dissemination of HIV. T cells can exit peripheral lymph nodes via the efferent lymphatics to enter other lymphoid tissues and the blood system [110,111]. By blocking T cell egress via administration of the functional sphingosine 1-phosphate receptor antagonist FTY720 at the time of infection, peripheral blood HIV RNA levels decreased to background levels. Drug-induced lymphopenia also reduced the levels of viral RNA loads two months after infection in secondary lymphoid tissues such as mesenteric lymph nodes and the spleen. This study concludes that migratory T cells serve as a vehicle for systemic dissemination of HIV.

### 5.2. HIV Nef Interferes with T Cell Diapedesis for Lymph Node Homing In Vivo

Many viruses encode for accessory proteins that are essential for different steps of the viral infectious cycle within the host [112,113,114]. For example, viral proteins can counteract host restriction factors for efficient replication, modulate cell signaling pathways or degrade host proteins for evasion from adaptive and innate immunity. The HIV accessory factor Nef is a multifunctional protein that has been shown to modulate the activity, localization, and abundance of host cell proteins. In vitro experiments revealed that Nef can mediate downregulation of many surface proteins such as CD4 and MHC-I [115,116,117], counteract the host restriction factors SERINC-5 and -3 [118,119] and inhibit cellular motility and chemotaxis in vitro by disrupting actin turnover through direct interaction with the cellular kinase PAK2 [120,121,122].

Recently, the impact of Nef on cell migration in vivo within a mammalian host was analyzed using MP-IVM [123]. Specifically, the effect of Nef on the homing efficiency of T lymphocytes into lymph nodes was visualized. This recirculation of T cells into lymph nodes requires a functional actin network for extravasation through high endothelial venules (HEVs) and subsequent migration within tissue parenchyma. Isolated primary mouse CD4+ T cells were transduced in vitro for functional Nef-GFP expression and adoptively transferred into mice for functional characterization. Here, expression of HIV Nef in primary mouse CD4+ T cells caused a significant reduction in the homing efficiency of T lymphocytes into lymph nodes. Nef-expressing CD4+ T cells enriched inside HEVs and single cell analysis after MP-IVM demonstrate reduced motility of Nef-positive cells in and near HEVs. Expressing PAK2 binding-deficient Nef mutant (Nef F195A) suggests that Nef-mediated inhibition of extravasation involves actin remodeling via PAK2-dependent cofilin phosphorylation. Furthermore, quantitative analysis of CD4+ T cell migration within the lymph node parenchyma shows a less pronounced reduction in the interstitial motility of T cells that express Nef. Taken together, MP-IVM reveals that HIV-1 Nef efficiently interferes with diapedesis of T lymphocytes from HEVs into lymph nodes resulting in a reduced homing efficiency but moderate effects on parenchymal motility.

### 5.3. MLV Establishes Infection in Secondary Lymphoid Tissue by Trans-Infection and Exploits Virological Synapses for Cell-To-Cell Transmission

In vitro studies of retrovirus spread within a cell population revealed different strategies that are based on the following concepts [124,125]. Retroviruses such as HIV and MLV can disseminate from an infected cell by diffusion through the extracellular space or efficiently spread by direct contact between virus-presenting donor cells and target cells. Productively infected cells establish Env glycoprotein-dependent cell-cell contacts with uninfected cells for subsequent virus transmission. The macromolecular structure at the contact site is described by the concept of the virological synapse (VS). In addition, non-permissive cells can capture virus particles and transfer them to a target cell for infection. The cell-cell contact formed during *trans*-infection is termed infectious synapse and strictly depends on the presence of the Env glycoprotein on viral particles.

Recently, MP-IVM was used to study the mechanism of retrovirus spread in vivo within secondary lymphoid tissues by visualizing MLV-infected cells and MLV particles directly in living animals. In a first study, in vitro transduced B cells were adoptively transferred into mice for MP-IVM at the popliteal lymph nodes [30]. In contrast to other studies visualizing infected cells expressing cytoplasmic GFP after reporter virus infection, full-length MLV encoding for a capsid fusion with GFP (Gag-GFP) helped to analyze the subcellular distribution of a structural retroviral protein in vivo. MLV Gag-GFP revealed a polarized distribution in static MLV-infected cells indicating the existence of VSs. Like the in vitro counterparts, in vivo synapses are dependent on the viral envelope glycoprotein and are characterized by a prolonged polarization of the viral capsid to the cell-cell interface.

A subsequent in vivo study using MP-IVM revealed how lymph- and blood-derived MLV can establish infection at secondary lymphoid tissues such as lymph nodes and the spleen [31]. Resident macrophages at the subcapsular sinus (SCS) floor of the popliteal lymph nodes were shown to bind and concentrate lymph-derived MLV particles for *trans*-infection of target B1 cells. Capture of fluorescent MLV Gag-GFP particles was shown to be mediated by an interaction between the lectin CD169 expressed by SCS macrophages and gangliosides within the retroviral envelope. Adoptively transferred B1 cells can engage in transient adhesive interactions with MLV-presenting macrophages. Although capture of retroviral particles was independent of the Env glycoprotein, reduced motility of B1 target cells and the formation of stable interactions with macrophages for virus transfer to target cells were mediated by Env. Subsequently, MLV-infected B1 cells formed Env-dependent VS with susceptible cells in vivo to amplify the infection.

In summary, MP-IVM studies of MLV within its natural host reveals cell-cell contacts leading to the formation of stable Env glycoprotein-dependent virological or infectious synapses. Since free viral particles are readily detectable in serum of mice during infection the studies suggest that multiple pathways of MLV transmission are evident in vivo strictly depending on the physiological context of the tissue [111].

### 5.4. Contact-Dependent Transmission Contributes to Local Tissue Spread of HIV for Multi-Copy Transmission of Viral Genomes

Contact-dependent transmission between cells was observed in vitro for many enveloped viruses [125]. Efficient spread from retrovirus-infected cells to target cells across virological synapses and *trans*-infection of target cells by virus-presenting cells have been described in vitro for HIV and MLV [47,48,49,126,127,128]. Recently, VS and *trans*-infection events could be observed for MLV in vivo suggesting that both processes can contribute to retroviral spread in vivo [30,31]. Further, in vitro studies revealed that cell-cell transmission of HIV results in the frequent co-transmission of multiple copies of the HIV genome across VS [129,130,131]. Together with the spatially high concentration of viral factors at contact sites cell-cell transmission leads to a reduced sensitivity of HIV to antiretroviral drugs [132,133] and resistance to certain broadly neutralizing antibodies [130,134,135,136].

A recent study challenged the concept of multiple copy transmission of HIV genomes under in vivo conditions [137]. The frequency of HIV co-transmission events and the spatiotemporal distribution of infected cells in the spleen of humanized mice were analyzed. After co-infection with two viral genomes high levels of co-transmission could be observed in humanized mice resulting in micro-anatomical clusters of the viral genomes within lymphoid tissue. Since the two HIV genomes encoded for distinct fluorescent proteins, spatial compartmentalization of HIV within the spleen could be visualized and quantified using MP-IVM. Computational modeling of the observed HIV spread in vivo supports a model for local viral replication of infected cells rather than spread by virus particle diffusion.

In addition, MP-IVM of HIV-infected cells in the spleen of humanized mice showed a significant reduction in the mean migration velocity. Quantitative analysis of infected and non-infected cells within the spleen of HIV-infected humanized mice demonstrates the formation of stable contacts between HIV-infected cells and target CD4+ T cells. The migratory arrest of target cells is induced by the Env glycoprotein expressed on the surface of HIV-infected cells. This observation confirms Env-dependent formation of stable cell-cell contacts observed for MLV infection in vivo [30,31]. A subpopulation of HIV-infected cells in the spleen of humanized mice revealed an elongated morphology because of syncytia formation confirming recent observations from HIV-infected human CD4+ T cells in peripheral lymph nodes of humanized mice [62]. Unfortunately, the role of cellular syncytia for the replication and pathogenesis of HIV infection is still elusive and needs to be determined in vivo [138].

### 5.5. PRV-Induced Changes in Ca^2+^ Signaling Causes Peripheral Neuropathy

Members of the Herpesviridae family include pathogens of the mammalian nervous system with characteristic latent, recurring infection. The Alphaherpesviridae subfamily contains important human-pathogenic viruses such as herpes simplex virus-1 (HSV-1) and varicella zoster virus (VZV) that can invade the peripheral and central nervous system of the host. Another member of this subfamily Pseudorabies virus (PRV) has been used for neuronal circuit tracing to visualize synaptically connected neurons in mammalian hosts [139] but also serves as a model to study infection of neurotropic viruses. PRV infection in mice shows symptoms of acute peripheral neuropathy with manifestations of peripheral pain or itching that result in self-mutilation due to violent scratching and biting [140]. Because PRV strains can tolerate the integration of genetic material to express fluorescent and reporter proteins, replication-competent PRV has been extensively used for in vitro live cell microscopy to study crucial steps of the virus life cycle such as virus particle transport within neurons [141,142], virus transmission across synaptic contacts and genome diversity following cell-contact dependent transmission [52]. Recently, a circuit-tracing PRV encoding for the calcium indicator protein GCaMP2 was used to monitor activity of neuronal circuits by measuring calcium flux in vivo using MP-IVM [143]. A follow-up study of the same group used MP-IVM to analyze neuronal activity during virulent PRV infection [144]. Imaging peripheral nervous system ganglia infected with PRV reporter virus encoding the calcium indicator protein GCaMP3 revealed changes in Ca^2+^ flux. PRV-infected submandibular ganglia flashed synchronously and cyclically in highly correlated patterns. Electrical coupling of axons in vivo required glycoprotein B and the viral membrane protein US9 suggesting that fusion proteins contribute to peripheral neuropathy of PRV infection without signs of fusion between ganglia.

## 6. Combinations of MP-IVM

MP-IVM allows minimal invasive studies of viral infections in real-time at cellular and subcellular resolution. Because of the intrinsic optical sectioning effect multi-photon excitation enables 3D imaging of cellular dynamics and even the diffraction-limited visualization of fluorescent viral particles in vivo. Despite its advantages in dynamic in vivo imaging applications, the technical and photophysical properties of multi-photon excitation limit the analysis with respect to image resolution, acquisition time and specimen volume. In addition, valuable parameters such as the transcriptional profile of host cells or high-resolution analysis of individual events within the imaged specimen cannot be determined by standard microscopy approaches using multi-photon excitation. To overcome these limitations and to allow a more detailed analysis within the same experiment, efforts were made to combine MP-IVM with other techniques and correlate the dynamic information of intravital imaging experiments with additional data sets. For example, intravital correlative light-electron microscopy (CLEM), the combination of MP-IVM with electron microscopy, revealed ultrastructural details of dynamic events in vivo (for review see [145]. In this section, additional combinations of MP-IVM with optogenetics and transcriptional analysis of single cells are summarized.

### 6.1. Optogenetic Manipulations of Protein Function and Cell Dynamics Using MP-IVM

Two-photon microscopy is the preferred approach for in vivo imaging studies because of reduced phototoxicity and increased imaging depth due to red shifted light for fluorophore excitation. Because of the intrinsic optical sectioning effect with selective excitation at the plane of focus, two-photon microscopy has the unique capability to initiate highly localized photochemical reactions. Photochemical processes such as photo-uncaging and photo-conversion can be specifically triggered under in vivo conditions with spatial and temporal precision.

The controlled manipulation of protein activity with a pulse of laser light is defined as optogenetics and bridges imaging approaches to a functional level. Originally limited to light-sensitive ion channel proteins such as the algal protein channelrhodopsin-2 used in the field of neurobiology to manipulate ion flux in mammalian neurons [146], the toolbox of photosensitive modules has been expanded tremendously in recent years (For review see [147,148]). Non-channel optogenetics is based on natural photoreceptors with chromophores for photon absorption leading to conformational changes of the receptor. Non-channel, light-controlled modules have been used to manipulate signaling cascades, stimulate DNA binding, alter cell polarity, and trigger cell motility with high temporal and spatial precision.

Biological processes in multicellular organism highly depend on the timing, location, and intensity of input signals. Multiple cues such as cell polarity, cytoskeletal dynamic, cell adhesion and cellular signaling must be coordinated to exert a specific function. For example, cell migration depends on front-rear polarity that is established by directional cues such as gradients of growth factors, chemokines, and ECM components [149,150,151]. Recently, the small GTPase Rac was combined with a flavin chromophore-containing light-oxygen-voltage (LOV) protein to artificially induce protein activation and membrane-recruitment of Rac1 to study cell migration [152]. LOV and light-activated dimerization domains respond to blue light (~400–500 nm) and can be used to control protein activity for manipulation of cell function reversibly and repeatedly. Photoactivatable Rac1 enabled an optical manipulation of membrane ruffling in living cells [152,153] and within living animals to direct collective cell migration in drosophila [154] and neutrophil motility in the zebrafish model [155]. Other photoactivatable variants of Rho-family small GTPases are available that function by oligomerization or localization of GTPases and GEFs to membranes [156,157,158,159,160]. In addition, LOV domains can be used to cage bioactive peptides by steric inhibition of protein binding sites for the control of endogenous protein activity. For example, fragments of formin autoinhibitory domains that activate endogenous mDia1 and a myosin light chain kinase inhibitor have been caged using this approach [161]. Other experimental systems might be used to modulate protein function that are of potential interest for in vivo studies of viral infections.

### 6.2. In Vivo Imaging and Transcription Analysis of Single Cells within Spatially Confined Tissue Using Photoactivation

Cellular functions in vivo are strongly dependent on the surrounding tissue environment. Cell composition and tissue-specific factors create a highly structured and spatially organized environment that is critical for the physiological function of the tissue and cells within. MP-IVM enables the visualization of defined cell populations or viral factors using fluorescent proteins and tags. Unfortunately, the surrounding space including cell populations remains invisible leaving a gap of knowledge of the microenvironment. Recently, a new approach was developed to define cellular and molecular composition of niches within the tissue of interest [162]. The method uses fluorescent reporters that are photo-activated within a spatially defined volume by light using MP-IVM. Subsequent sorting of cells with flow cytometry allows single-cell RNA-seq analysis of cells within the tissue microenvironment. Thus, niche-specific analysis of rare cell subpopulations and unique gene programs of cells can be analyzed and set into the defined tissue context. This novel approach represents a powerful method to study local events during virus infection since immune function but also virus pathogenesis depends on the dynamic interaction of heterogeneous cell types within the tissue microenvironment. For example, macrophages exhibit organ-specific cellular functions due to interactions with distinct cells in different tissues, but also tissue-resident immune cells are shaped by the specific tissue environment. In the study the cellular composition of lymph node B cell follicles and the T cell area after LCMV infection was analyzed in a mouse model. This powerful approach will be very interesting for future studies of viral infections in vivo.

## 7. Summary and Future Perspectives

Intravital microscopy using multi-photon excitation is a powerful technique to visualize viral infections in living organisms. Reporter viruses for cytoplasmic GFP expression have been used to study the dynamic behavior of retrovirus-infected cells in vivo, revealing important insights into the mechanism of how viruses establish infection, spread within target organs, and disseminate systemically in the host organism [30,31,62]. In addition, first details about the pathogenesis of PRV and HIV have been analyzed in animal models using MP-IVM [123,144]. The effects of PRV infection on neuronal signaling could be visualized after expression of calcium indicators in infected neurons and HIV Nef-mediated inhibition of *trans*-endothelial migration of infected CD4+ T cells. The model retrovirus MLV has even been used to directly visualize retroviral particles in vivo by fusion of GFP to the MLV capsid protein Gag [30,31]. MP-IVM studies revealed the formation and dynamic of virological synapses formed by MLV Gag-GFP-infected B cells and allowed researchers to visualize the capture of lymph-derived MLV particles by macrophages for *trans*-infection of target cells in real-time. For future studies, it will be exciting to visualize and quantify the spatial and temporal dynamics of other viral proteins. In particular, the subcellular distribution of virus structural proteins, glycoproteins, and accessory proteins such as Nef and Vpr from HIV during virus spread will be of great interest.

The visualization of subcellular compartments and host cell proteins during infection is going to be another critical aspect for MP-IVM studies of viral pathogens. For example, imaging of cell adhesion proteins, restriction factors, factors of the immune response and cell signaling proteins will provide critical insights into viral infections and host immune responses. For example, activation of the inflammasome complex in SCS macrophages as response to modified vaccinia Ankara virus infection was observed in vivo using MP-IVM [163]. Further, CD8+ T cell-triggered apoptosis of tumor cells and apoptosis-mediated contraction of CD4+ T cells could be visualized in vivo using an apoptosis reporter consisting of a FRET-based reporter for caspase-3 activity [164,165]. Similar approaches could be helpful to study HIV-induced bystander cell death in a humanized mouse model. In addition, visualizing collagen by label-free SHG could be a valuable tool to study lymphoid tissue fibrosis in response to HIV infection. Extensive deposition of collagen in lymph nodes has been proposed to contribute to the depletion of CD4+ T cells by apoptosis during chronic HIV and SIV infection by limiting physical access to survival factors such as IL-7 [166,167].

Up to now, visualizing cell signaling in vivo has been done in the field of neurobiology and immunology. Subcellular distribution and the dynamics of the signaling protein linker for activation of T cells (LAT) during the formation of immunological synapses and the atypical protein kinase C zeta to analyze anteroposterior polarity in migrating cells has been analyzed in vivo [168,169]. In addition, Ca^2+^ signaling in lymphocytes during migration and during cell-cell contact formation for immune priming has been visualized using FRET-based calcium biosensors [170]. Further, the subcellular redistribution of transcription factors such as NFAT1 in response to cell-cell could be analyzed in vivo [171,172,173]. A first report using MP-IVM started to address the effects of virus infection on cellular signaling. Calcium signaling in PRV-infected neurons has been visualized using genetically encoded Ca^2+^ indicator such as GCamP3. Since activation of cell signaling networks at immune cell contacts was recently shown to be essential for signaling-dependent enhancement of HIV dissemination in vitro [174], MP-IVM of cell signal proteins during viral infection in vivo is another exciting research field for future studies.
